# Is climate change hindering the economic progress of Nigerian economy? Insights from dynamic models

**DOI:** 10.1016/j.heliyon.2024.e39288

**Published:** 2024-10-11

**Authors:** Sodiq Arogundade, Adewale Samuel Hassan, Biyase Mduduzi

**Affiliations:** College of Business and Economics, University of Johannesburg, Auckland Park Kingsway Campus PO Box 524 Auckland Park, Johannesburg, South Africa

**Keywords:** Climate change, Economic growth, ARDL, Quantile ARDL, FMOLS, Time-varying Granger-causality causality test, Nigeria

## Abstract

This study investigates the link between climate change and economic growth in Nigeria from 1961 to 2022. To provide a robust analysis that facilitates a nuanced examination of this dynamic relationship, this study employs state-of-the-art econometric approaches, including autoregressive distributed lag (ARDL), fully modified least squares (FMOLS), novel quantile autoregressive distributed lag (QARDL), and time-varying causality. The empirical results of this study are as follows: (1) the impact of climate change on economic growth is not felt in the short run. However, climate change negatively influences economic growth in Nigeria in the long run, (2) the elasticity of climate change increases across the conditional quantile economic growth, (3) unidirectional causality from climate change to economic growth across different time dimensions. These empirical outcomes advocate for a proactive and adaptive policy framework, emphasising the need for the Nigerian government to adopt climate-smart policies.

## 1 Introduction

Without a doubt, climate change is already occurring widely, and because of the serious risks it poses to the environment, human welfare, and development, it is recognised as the biggest problem of the twenty-first century^1^,^2^ Climate change is mainly caused by greenhouse gases released into the atmosphere by human activity. These gases are also the main cause of global warming. According to Refs. [1,2], growing carbon dioxide emissions are unquestionably dangerous for the planet's future, endangering both long-term human existence and environmental sustainability. Climate change poses significant challenges for Nigeria due to its impacts on various sectors, including agriculture, water resources, health, and overall economic stability [[3], [4], [5], [6], [7], [8]] Nigeria's economy heavily relies on agriculture, and climate change affects rainfall patterns, leading to droughts and flooding, which in turn disrupt agricultural productivity. According to Ref. [9], Nigerian farmers' net farm income is affected by even minor fluctuations in temperature and precipitation.

Projections for the future indicate that the climate will continue to change for the rest of this century and beyond [10]. The COP26^3^ (2021) report also demonstrated the worsening state of affairs, with the most recent revised prediction pointing to a notable 13.7 % increase in global greenhouse gas emissions in 2030 over 2010 levels. Moreover, the IPCC estimates that CO_2_ emissions need to be cut by 45 % and 25 % by 2030 to keep global warming from 1.5 °C to 2 °C. The situation may get more challenging and complex due to this growing trend [11]. As a result, during the UN Climate Change Conference, COP26, in Glasgow, policymakers continued to place a high priority on climate change.

Since the 20th century, the international community has prioritised climate change and signed numerous agreements to cut greenhouse gas emissions and safeguard the environment because of its socioeconomic costs. This includes the Paris Agreement, the Kyoto Protocol, and the United Nations Framework Convention on Climate Change [11]. One of the main challenges facing social progress and economic expansion is climate change, and studies have shown that it is the primary cause of increased food risks and reduced agricultural yield [12,13], worsening disease incidence [14], and wealth inequality [15].

Nigeria's economic growth has gone through different phases over the past decades. From 2000 to 2014, Nigeria's economy achieved consistent and widespread growth, averaging over 7 % yearly. According to the [16], this growth was driven by favourable global conditions, effective macroeconomic policies, and first-stage structural reforms. On the other hand, the economy experienced declining growth rates for the period 2015–2022 [16]. During this period, GDP growth struggled to match the pace of population growth, leading to a gradual decrease in per capita income. Additionally, the country consistently fell below the sub-Saharan African average on all significant measures of health and well-being, including the World Bank's Human Capital Index [16,17].

The [16] attributed the Nigerian economy's declining growth rates in the last decade to a few factors, one of which was external shocks. Climate change could be a profound and multifaceted external shock to economic growth [18], as climate change-induced severe weather events like hurricanes, floods, and droughts can cause substantial economic damage, affecting agriculture, industry, and livelihoods. Its environmental impact can lead to loss of biodiversity, degradation of ecosystems, and increased frequency of natural disasters, with concomitant effects on water supplies, agriculture, and natural habitats [[19], [20], [21]]. Considering Nigeria's economic and environmental attributes makes it an intriguing focus of study on the impact of climate change on economic growth.

Nigeria is home to abundant natural resources, especially oil and natural gas [22]. Understanding the impacts of climate change on Nigeria's economy can provide insights into how such a significant economy is vulnerable to climate-related risks and what mitigation strategies might be effective. For example, the incessant dredging of major rivers, flaring of gas, reclaiming land for oil and gas extraction, oil spills, and associated activities undertaken by oil companies have caused significant environmental deterioration in the Niger Delta region of the nation, with its economic toll estimated at approximately US$758 million annually [23]. According to Ref. [24], local communities shoulder approximately 75 % of this substantial burden through effects such as infertile farmland, reduced biodiversity, diminished harvests, and contaminated water. The indirect effect of this on economic growth needs to be validated.

Nigeria is one of Africa's top-four economies and the most populous country [16]. Its numerous ecological zones have produced diverse commodities, farming techniques, and livelihoods that are all impacted by shocks and changes in the climate. Rising sea levels put coastal communities and towns in the south, like Lagos, at risk of floods and waterborne illness. Rising temperatures, drought and less rainfall, impede the nation's hydropower systems, hamper fishing and agricultural productivity, lower food security, and harm nutrition and health [25]. Deforestation, land-use change, and the energy industry are the main causes of Nigeria's greenhouse gas (GHG) emissions [26].

**Fig. 1:**
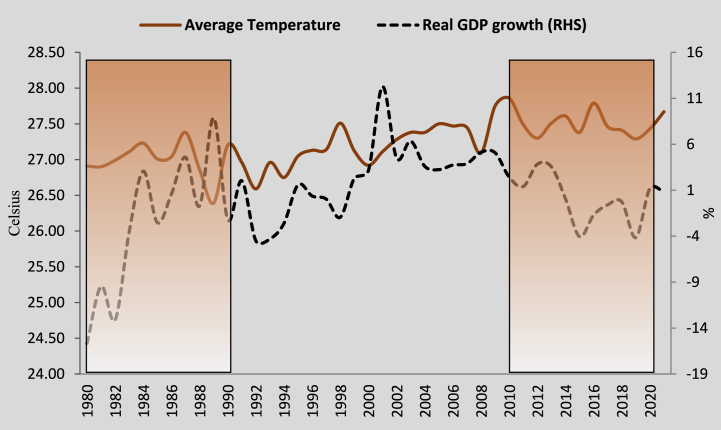
Trend of annual average temperature versus real GDP growth in Nigeria.

This study's objective is to examine the linkage between climate change and economic growth in Nigeria. In Nigeria, climate disasters are occurring at frightening rates. Flooding claimed the lives of at least 662 people, wounded 3,174, caused nearly 2.5 million to be homeless, and destroyed 200,000 homes and persons in 2022 alone [27]. Furthermore, Fig. 1 shows the impact of annual average temperature changes on the economic growth rate. It can be seen that whenever the average temperature increases, economic growth declines. For instance, a 7.31 % and 4.19 % growth in average temperature from 1988 to 1998 and 2011–2021 corresponds to a decline in economic growth by −23.34 % and −1.065 %, respectively. The conjecture that climate change reduces economic growth in Nigeria, as presented in Fig. 1, cannot be relied on, as it has not been backed by empirical evidence, leaving room for further research and experimental validation.

Source [40,41].

Several attempts have been made to examine the link between climate change and economic growth, but the findings are inconclusive. While studies such as [28,29], and [30] argued that climate change deteriorates economic growth. Other studies established a non-linear relationship between climate change and the economy [8,31]. Moreover, some studies have determined a specific threshold at which its impact becomes negative on the economy [32,33]. At the same time, it was also established that compared to richer countries, poorer countries are far more vulnerable [34]. Nonetheless, most of these studies were conducted on a panel of countries without considering country-specific characteristics.

The novelty of this study can be summarised as follows. First, this study contributes to the existing literature by analysing the effect of climate change on economic growth across various quantiles of economic growth. This enables the investigation of this effect across various levels of economic growth, specifically when economic growth is relatively high, average and quite low. To this end, we used an advanced version of the ARDL model known as the quantile ARDL (QARDL) model, introduced by Ref. [35]. We also employed ARDL and FMOLS approaches for robustness and compared their estimates with those from the advanced QARDL model. Second, we used sophisticated Ganger causality tests in Refs. [36,37] tests in assessing the time-varying Granger causality between climate change and economic growth. This approach enables the possibility of observing how causation evolves over time [38]. Third, this study adds to the existing literature by focusing on Nigeria, which has been previously ignored by studies exploring the climate change-economic growth nexus despite being described as a zone severely impacted by climate change [39].

This study is structured as follows. Section 1 introduces the study and provides stylised facts regarding climate patterns in Nigeria. Section 2 encompasses a review of the literature, while Section 3 discusses the methodology. Section 4 contains the presentation, interpretation and discussion of empirical results, and Section 5 concludes the study.

### 1.1 Climate pattern in Nigeria

Nigeria has been identified as a climate change hotspot due to its heightened vulnerability to drought, floods, and irregular rainfall patterns [39]. Nigeria is home to three distinct climatic zones: a hot, semi-arid Sahelian climate in the north, a tropical monsoon climate in the south, and a tropical savannah climate in the majority of the country's middle areas.^4^ As a result, precipitation gradients from the south to the north have decreased. The southern parts get periods of intense rainfall from March to October when the rainy season occurs. In the Niger Delta, annual rainfall quantities usually surpass 4,000 mm^3^.

The rainy season (April to September) and the dry season (December to March) are the two common seasons in the central region. The dry season is influenced by the Harmattan wind from the Sahara. In coastal places with a brief dry season, the majority of the rainy season occurs from March to October. From June to September, the north experiences just 500–750 mm of rain each month. The rest of the year is dry and hot; in the north, sharp fluctuations in the yearly rainfall pattern result in both flooding and droughts.

**Fig. 2:**
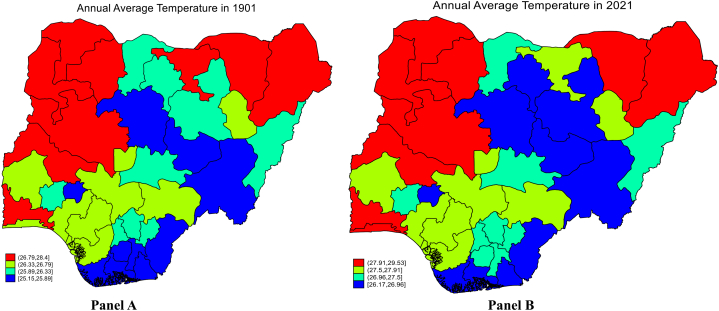
Pattern of Nigeria's average temperature.

Nigeria has an average yearly temperature of 26.9 °C, with average monthly temperatures ranging from 24 °C in December and January to 30 °C in April. Precipitation averages 1,165.0 mm per year ([41]. Nigeria experiences rain every month of the year, with April through October seeing the most substantial amounts and November through March seeing the least amount. However, how can you account for harmattan in February/March or Taraba State's “snowfall”? Even severe drought in areas of rainforests? These scenarios are linked to climate change. Fig. 2 (Panel A and B) also bolsters the argument of climate change in Nigeria. The average temperature across all 37 states in 1901 is 26.42 °C and 27.52 °C in 2021.

Source [41].

## 2 Literature review

### 2.1 How does climate change influence the economy?

**Fig. 3:**
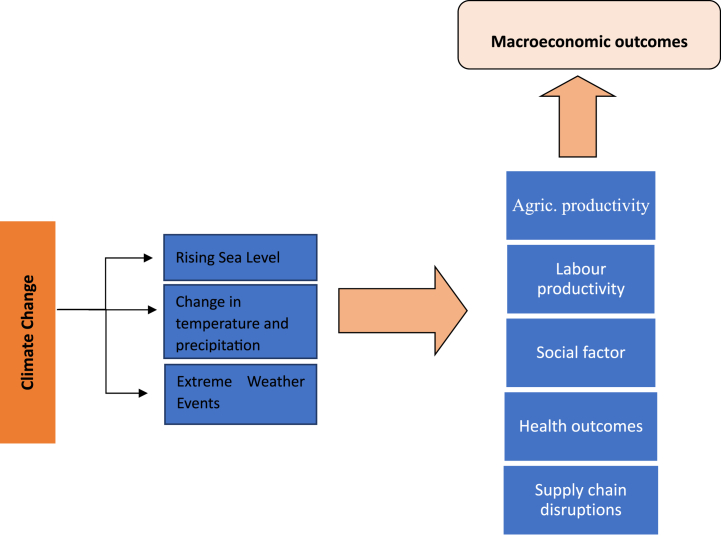
Transmission mechanism of climate change on economic performance.

Source: Authors' extract from the literature.

Fig. 3 presents the transmission mechanism of how climate change influences the economy. This figure is drawn based on the proposition of the literature. Climate change, which comes in the form of rising sea levels, water scarcity, changes in the pattern of precipitation and temperature, and extreme weather conditions like floods, hurricanes, and droughts, has the potential to affect the supply chain by damaging transportation infrastructure and interrupting the production and distribution of goods and services [42].

Similarly [43], argued that the health impacts may include gastrointestinal illness like diarrhoea, effects on the body's nervous and respiratory systems, or liver and kidney damage. High temperatures can also trigger social unrest, conflict and violence brought on by famine [44]. This also increases emigration as people seek greener pastures [45]. Studies like the [46,47] have argued that climate change is responsible for the herder-farmers crisis in Nigeria.

The impact of climate change on labour productivity has also been documented by Refs. [48,49]. These studies argue that the human body responds to rising temperatures and humidity levels in the environment by raising blood flow to the skin's surface and producing perspiration to regulate the body's internal temperature. There may be a drop in thermal comfort and an increase in difficulty with both physical and mental duties. Consequently, labour productivity begins to drop. All of the aforementioned impacts of climate change lead to an increase in food prices and a reduction in aggregate supply and demand, potentially impacting inflationary pressure and aggregate output reduction.

### 2.2 Climate change and economic growth – brief empirical literature

Earlier literature on the link between climate change and the economy has focused more on the deleterious impact of the economy on the environment and climate change [50]. tested the EKC hypothesis and showed that following an initial beneficial impact, increased economic growth exacerbates climate change in Africa through its negative effects on environmental quality. In a similar study [51], validated the EKC for China, where GDP initially enhanced carbon efficiency before reaching a level where more income decreased carbon efficiency. Several studies [[52], [53], [54], [55], [56], [57]] also demonstrated that economic growth exerts a damaging effect on the environment and climate. Furthermore [58], revealed that the economic growth of mineral resources in China is linked to increased global warming and environmental pollution [59]. provided evidence of a cross-correlation between GDP and climate change.

Meanwhile, emerging literature is now beginning to examine how climate change influences macroeconomic performance—starting with the study of [30], which examined the impact of climate change on the economic growth of selected Asian countries. Using the GMM estimation method and augmented Solow growth model framework, the study discovered that climate change deteriorates Asian output per worker. A meta-analysis by Ref. [34] resulted in some interesting findings. First, it was found that the central estimate of global warming's economic effects is consistently negative. Second, it demonstrated that the uncertainty surrounding the impact of climate change is skewed towards unfavourable shocks. It also revealed that compared to affluent countries, poorer countries are far more vulnerable. Moreover, a study on Nigeria using multiple regression and the Cobb-Douglas test [6] demonstrated that climate change has placed numerous constraints on human development, which has negatively impacted Nigeria's environment and economic growth.

Similarly [60], discovered that achieving climate-change mitigation targets will substantially decrease economic damage [33]. also found that limiting warming to 1.5 °C would minimise economic damages relative to 2 °C by the end of this century with a more than 75 % chance and that the accumulated global benefits under a 3 % discount rate (2010 US dollars) will exceed US$20 trillion with a more than 60 % chance. The study further suggested that achieving the 1.5 °C target will likely reduce aggregate damage and lessen global inequality and that failing to meet the 2 °C target will substantially increase economic damage. In their empirical study [29], indicated that GDP growth reduces by 0.667 percentage points for every one-unit increase in temperature in Africa. The study alluded to the fact that temperature's impact is heterogeneous across the 34 African countries. Additionally [61], confirms most of the literature's position that temperature negatively impacts economic growth. However, this impact is conditional on the readiness and adaption of these countries.

[32] used country-level data for Africa to analyse the convergence of climate change damage and integrated an economic model with three risk dimensions. They discovered that the damage caused by climate change accounted for 10–15 % of GDP per capita growth and that East African countries are expected to have challenges in adapting to the changing climate. In a similar study [28], used quantile regressions to investigate the effects of climate change on the risks associated with economic activity. Estimates from the study revealed that temperature has a significant and robust impact across specifications on the risks to economic growth.

However, some studies have argued a nonlinear relationship between climate change and economic growth. Using the pooled mean group (PMG) estimator and two climate variables: precipitation and temperature [31], established that temperature above 24.9 °C would significantly reduce African countries' economic performance. The study further established a nonlinear relationship between climate change and economic growth. Using the fixed effect method and the panel vector autoregression [8], examined the impact of climate change on the economic growth of African countries. The empirical outcomes demonstrate that temperature has an inverted U-shape impact on the economic performance of countries in tropical dry climate zones and rainforests. Nonetheless, a U-shape relationship was observed in the warm temperate humid regions.

The literature is replete with unremitting debate on the link between climate change and economic growth. While the majority argue that climate change has a deteriorating impact, others support the view that the impact is nonlinear. Some studies also indicate a specific threshold at which its impact becomes negative on the economy. However, the majority of these studies have been done as a group (Africa, the world, Asia, and Europe) without considering a country-specific situation. This study assesses whether climate change contributes to the fragile and weak economic performance of the Nigerian economy. Methodologically, the study uses ARDL, FMOLS, Quantile ARDL and a time-varying causality.

## 3 Methodology

### 3.1 Theoretical framework

This investigation of the linkage between climate change and the economic growth of Nigeria is theoretically predicated on the neo-classical growth model of [62], which examines how to achieve economic growth through the combination of three fundamental engines of growth: labour, capital, and total factor productivity (or technology). More specifically, this study follows previous empirical studies [29,63,64], which put forward a version of the [62] model that incorporates climatic variables.

The [62] model assumes that economic agents combine labour and capital to produce output in an economy and that the overall output model for the economy is as follows:

(1) $$Y={AK}^{\alpha }L^{\beta }$$

where Y is real output, K is stock of capital, L is labour, A is total factor productivity (TFP) or technology, while α and β are elasticities of output for capital and labour, respectively.

The underlying assumptions of equation (1) are characterised by constant returns to scale, the first- and second-order derivatives of its intensive form satisfying the Inada conditions, and that the growth rate of TFP or technology at a constant rate is g in equation (2) as follows:

(2) $$A=A_{0}e^{gt}$$

In line with [63], $A_{0}$ stands for country-specific elements like institutions, natural resources, and climate. The components included in this parameter should, however, become a part of the dynamic production function if at least one segment of those components is not constant over a long period of time. In light of this, model climate variability is modelled with the incorporation of climate change into the model under the assumption of a growth-drag effect:

(3) $$A_{t}=\varphi {CC\left(t\right)}^{\sigma }$$

where φ is a time-invariant constant, CC is climate change with proxies as temperature and precipitation [65]. By accounting for equation (3) in equation (1), we have:

(4) $$Y\left(t\right)=\varphi {CC\left(t\right)}^{\sigma }{K\left(t\right)}^{\alpha }{L\left(t\right)}^{\beta }$$

Equation (4) is transformed logarithmically and converted into an empirical model that incorporates other drivers of economic growth as follows:

(5) $$\ln\;{GDP}_{t}=\alpha_{0}+\alpha_{1}\;\ln\;{CC}_{t}+\alpha_{2}\;\ln\;K_{t}+\alpha_{3}\;\ln\;L_{t}+\alpha_{4}\;\ln\;{FD}_{t}+\alpha_{5}\;\ln\;{GL}_{t}+\varepsilon_{t}$$

where GDP is real GDP per capita, representing economic growth, CC is climate change measured by average temperature, as well as precipitation, K is stock of physical capital measured by gross capital formation, L is labour force measured by the level of labour force participation, FD is financial development measured by credit to the private sector (CPS), GL is globalisation measured by KOF Globalisation Index and $\varepsilon_{t}$ is the error term.

### 3.2 Methodology and data

The autoregressive distributed lag (ARDL) or bound testing technique of [66] is used to estimate the growth model. This estimation method is highly rated among researchers because of its various merits. It can generate reliable estimates in models containing variables that are entirely I(0) or I(1) processes or a combination of both I(0) and (1) processes. Therefore, difficulties regarding the level of integration are avoided. Also, a model's long-run and short-run parameters can be estimated simultaneously using the ARDL technique. Moreover, the endogeneity issue can be satisfactorily addressed using the method. Equation (5) is thus stated in ARDL form as follows:

$${\Delta GDP}_{t}=\theta +\sum_{i=1}^{p}\emptyset_{1}{\Delta GDP}_{t-i}+\sum_{i=1}^{p}\emptyset_{2}{\Delta CC}_{t-i}+\sum_{i=1}^{p}\emptyset_{3}{\Delta K}_{t-i}+\sum_{i=1}^{p}\emptyset_{4}\Delta L_{t-i}+$$

(6) $$\sum_{i=1}^{p}\emptyset_{5}{\Delta FD}_{t-i}+\sum_{i=1}^{p}\emptyset_{6}\Delta {GL}_{t-i}+\vartheta_{1}{GDP}_{t-1}+\vartheta_{2}{CC}_{t-1}+\vartheta_{3}K_{t-1}+\vartheta_{4}L_{t-1}+\vartheta_{5}{FD}_{t-1}+\vartheta_{6}{GL}_{t-1}+\varepsilon_{t}$$

Within the framework of the ARDL technique, the null of no cointegration among the variables ($H_{0}:\emptyset_{1}=\emptyset_{2}=\emptyset_{3}=\emptyset_{4}=\emptyset_{5}=\emptyset_{6}=0)$ is tested against the alternative hypothesis that the variables are related in the long run $\left(H_{1}:\vartheta_{1}\neq \vartheta_{2}\neq \vartheta_{3}\neq \vartheta_{4}\neq \vartheta_{5}\neq \vartheta_{6}\neq 0\right)$. The joint significance of the estimated coefficients of the lag level, from which the F-statistic is generated, is tested for the long-run relationship among the variables in equation (6). Following that, the computed F-statistic is placed up against the two critical upper bound and lower bound values that [66] provided. The null hypothesis that there is no long-run association between the variables is rejected if the estimated F-statistic exceeds the upper bound critical value. On the other hand, the null hypothesis that there is no long-term link between the variables cannot be rejected if the estimated F-statistic is smaller than the lower bound critical value. The calculated F-statistic is inconclusive if it lies between the lower bound and upper bound critical values. In this case, the result of the error-correction term would determine whether there is cointegration among the variables.

The robustness of the results is tested with fully modified least squares (FMOLS) regression, which has received acclaim in the literature over its ability to produce reliable estimates even when endogeneity, simultaneity and serial correlation are present [67,68]. With FMOLS, long-run estimation is enabled if there is cointegration among the variables [69]. According to Ref. [70], FMOLS regression is the best-suited regression for cointegrated series.

Due to the limitations of traditional ARDL and FMOLS models, this study used the Quantile ARDL model. Quantile regressions are used to estimate the conditional median or a variety of different quantiles of the response variables, as opposed to regular least-squares regressions, which produce estimates of the conditional mean of the endogenous variable subject to specific values of the exogenous variables [35]. Equation (6) is transformed thus as:

(7) $${GDP}_{t}=\alpha_{0}\left(\tau \right)+\sum_{i=0}^{q-1}W_{t-1}\delta_{j}\left(\tau \right)+X_{t}\gamma \left(\tau \right)+\sum_{i=0}^{q}\varphi_{i}\left(\tau \right){GDP}_{t-1}+U_{t}$$

In equation (7), (τ) is expressed as $\sum_{i=0}^{q-1}W_{t-j}\theta_{j}\left(\tau \right),W_{t}=\Delta X_{t}$, and $\delta_{j}\left(\tau \right)=-\sum_{i=0}^{p}*\phi_{i}\left(\tau \right)X_{t-i}$. The conditional mean function of GDP on climate change variable is estimated in equation (8):

(8) $$\frac{\min}{\beta }[\theta \sum {GDP}_{t}-X_{t}\beta +\left(1+\theta \right)\sum {GDP}_{t}-X_{t}\beta \{t\text{:}{FS}_{t}\geq X_{t}\beta \}\{t:{FS}_{t}<X_{t}\beta \}$$

{GDP,t=1,2…,T} is a random sample on the regression process. $Y=\alpha_{t}+X_{t}\beta$ with conditional distribution function of $F_{\frac{Y}{X}\;}\left(GDP\right)=F\left({GDP}_{t}\leq CC={GDP}_{t}-X_{t}\beta \right)$ and $\left\{X_{t},=1,2\;\ldots ,T\right\}$ is the sequences of (row) k-vectors of a known design matrix. The $\theta^{th}$ regression quantile, $Q_{\frac{GDP}{X}}\left(\theta \right),0<\theta <1$ 1 is any solution to minimise problems, $\beta_{\theta }$ denotes the solution from which the $\theta^{th}$ conditional quantile $Q_{\frac{GDP}{X}}\left(\theta \right)=x\beta_{0}$. Once the estimates from the baseline QARDL regression are obtained, then the long-run estimator is given as follows in equation (9):

(9) $$1-\sum_{i=0}^{p}*\varphi_{i}{\left(\tau \right)}^{-1}$$

The short-run and error correction models are estimated as follows in equation (10):

(10) $${\Delta GDP}_{t}=\alpha_{0}\left(\tau \right)+{ℶ}_{*}\left(\tau \right)\left({GDP}_{t-i}-\beta \left(\tau \right)X_{t-i}\right)+\sum_{i=0}^{p-1}\varphi_{i}\left(\tau \right)\Delta {GDP}_{t-i}+\sum_{i=0}^{p}*\varphi_{i}\left(\tau \right)\Delta X_{t-i}+U_{t}$$

$\left({GDP}_{t-i}-\beta \left(\tau \right)X_{t-i}\right)$ is the quantile error correction term.

Lastly, in assessing the time-varying Granger Causality between climate change and economic growth in Nigeria, we explored [36,37] bivariate LA-VAR model for integrated variables. This model's appeal is that it allows us to observe how causation evolves over time [38].

#### 3.2.1 Data

Annual data for Nigeria, spanning 1961–2022, were employed in this study. The dependent variable is economic growth, and it is measured by real GDP per capita. The main explanatory variable is climate change. As in previous studies [31,65,71], climate change is measured by average temperature and precipitation, and they were sourced from the Climate Change Knowledge Portal of the World Bank. Other independent variables in the model are capita stock, labour, financial development, and globalisation, which are measured by gross capital formation, labour force participation, credit to the private sector, and the KOF Globalisation Index, respectively. Data for GDP per capita, gross capital formation, labour force participation and credit to the private sector were sourced from the World Bank's World Development Indicators. In contrast, data for the KOF Globalisation Index was collected from the KOF Swiss Economic Institute.

##### 3.2.1.1 Summary statistics of important variables

Table 1 presents the descriptive statistics of the important variables explored in this study. The average temperature of Nigeria from 1961 to 2022 is 24.74 °C. The highest temperature of 26.49 °C coincides with when the world witnessed the warmest decade on record [72]. However, Nigeria experienced the coolest temperature of 22.38 °C in 1983. Similarly, precipitation ranged from 887.75 to 1356.02, averaging 1173.28. Additionally, real GDP per capita has an average of $1874.39, and it ranges from $1223.53 to $2679.55. The Jarque-Bera statistics also suggest that most of the variables (PR, GDPPC, GCF, L) failed the normality test as they are statistically significant. The motivates for the use of quantile regression estimation in this empirical study.

**Table 1:** Descriptive statistics.

Variable	Obs	Mean	Std. Dev.	Min	Max	J-B
AT	61	24.79	0.907	22.38	26.49	3.995
PR	61	1173.288	91.022	887.75	1356.02	7.711∗∗∗
GDPPC	61	1874.399	430.077	1223.532	2679.555	5.28∗
GCF	61	9.056e+10	6.360e+10	1.235e+10	2.437e+11	5.036∗
L	61	46286190	16706499	31936585	87080564	6.34∗∗
KOFGI	61	42.869	9.596	29.519	57.227	4.58
CPS	61	8.65	3.326	3.704	19.626	1.92

NB: ∗, ∗∗ and ∗∗∗ indicate 10 %, 5 % and 1 % significance levels, respectively. Credit to the private sector (% of GDP), AT: Average Temperature, PR= Precipitation, GDPPC= Real GDP per capita, GCF = gross fixed capital (% of GDP), L = Total labour force, KOFGI = Globalization index.

## 4 Results and discussion

### 4.1 Unit root tests

To ensure the selection of the appropriate estimation technique, unit root tests of Augmented Dickey-Fuller (ADF) and Phillips-Perron (PP) have been conducted on the variables. The results are presented in Table 2, and they reveal that besides the log of GCF, all the variables (lnGDP, lnAT, lnPR, lnLABOUR, lnKOFGI and lnCPS) are confirmed by ADF and PP to be stationary at first difference. Meanwhile, ADF and PP unit root tests have reportedly been shown to have poor power in the event of a structural break in the series. Consequently, the [73] test, which accounts for structural breaks in series, has also been applied, and the results are presented in Table 3. The results affirm stationarity for all the variables at first difference.

**Table 2:** Augmented Dickey-Fuller and Phillips Perron unit root tests.

Variable	Augmented Dickey-Fuller		Phillips Perron	
	Level	1st Difference	Level	1st Difference
lnGDP	0.876	−5.081∗∗∗	0.792	−5.624∗∗∗
lnAT	0.105	−10.682∗∗∗	0.055	−11.892∗∗∗
lnPR	−0.47	−3.848∗∗∗	−0.190	−12.655∗∗∗
lnGCF	3.311∗∗∗	–	−3.251∗∗∗	–
lnLABOUR	3.368	4.099∗∗∗	6.904	9.719∗∗∗
lnKOFGI	2.188	2.988∗∗	2.188	−5.809∗∗∗
lnCPS	−0.16	−6.501∗∗∗	0.474	−8.809∗∗∗

Note: ∗∗ and ∗∗∗ indicate 5 % and 1 % levels of significance respectively.

**Table 3:** Zivot-Andrews unit root test.

Variable	Level		First difference	
	t-value	Break year	t-value	Break year
lnGDP	−1.092	1998	−5.822∗∗∗	2018
lnAT	−4.713	2011	−7.271∗∗∗	2014
lnPR	−2.166	2017	−5.962∗∗∗	2004
lnGCF	−1.384	2000	−8.974∗∗	1998
lnLABOUR	−3.115	2001	−6.210∗∗∗	2012
lnGLOB	−2.102	1997	−5.532∗∗	2003
lnCPS	−4.542	2013	−5.049∗∗	2000

Note: ∗∗ and ∗∗∗ indicate 5 % and 1 % significance levels, respectively.

### 4.2 Cointegration test

The tests of cointegration based on the ARDL bound testing are presented in Table 4. For the Temperature model, the F-statistic (4.64) exceeds the upper bound critical value (3.34) at a 5 % level of significance. Similarly, the F-statistic of the Precipitation model (3.64) is greater than the upper bound critical value (3.34) at the 5 % level. These results demonstrate a long-run association among all the variables in the two models.

**Table 4:** Bound test for cointegration.

	Temperature model	Precipitation model
F-Statistic	4.64∗∗∗	3.64∗∗
**Narayan (2005) Critical values**		
Level of significance	I(0)	I(1)
10 %	1.81	2.93
5 %	2.14	3.34
1 %	2.82	4.21

Notes: Null Hypothesis: No long-run relationship exists (no cointegration). The selection of the model (lags) is based on AIC. The bounds test critical values are from Ref. [67] Critical values. ∗∗∗ is significance at 1 %, ∗∗ is significance at 5 %.

### 4.3 Assessing the costs of climate change on Nigeria's economic growth

The long-run estimates of the variables are provided in Table 5 via the ARDL regressions and in Table 6 via the FMOLS regressions. Two ARDL regressions were conducted, with the first one having the average temperature (AT) as the measure of climate change and the main explanatory variable. In the second model, the main explanatory variable is the log of precipitation (PR), which measures climate change.

**Table 5:** ARDL estimation results.

Variable	Temperature model		Precipitation model	
	Coefficient	t-Statistic	Coefficient	t-Statistic
**Long-run results:**				
lnAT	−0.672∗∗	−2.159		
lnPR			−0.231∗	−1.719
lnGCF	0.201∗∗∗	2.845	−0.044	−1.105
lnLABOUR	0.396∗∗∗	4.161	0.170∗∗	2.167
lnKOFGI	0.591∗∗	2.640	−0.251	−1.089
lnCPS	0.099∗∗	2.615	0.055	1.318
**Short-run results:**				
ECT	−0.111∗∗∗	−5.799	−0.052∗∗∗	−5.059
D(lnAT)	0.258∗∗	2.185		
D(lnAT(-1))	0.465∗∗∗	3.359		
D(lnPR)			0.033	0.386
D(lnPR(-1))			0.362∗∗∗	3.873
D(lnPR(-2))			0.237∗∗∗	2.829
D(lnGCF)	0.200∗∗∗	4.066	−0.005	−0.084
D(lnGCF(-1))	0.143∗∗	2.746		
D(lnLABOUR)	−0.606∗∗	−2.156		
D(lnKOFGI)	−0.586∗∗∗	−3.128	0.049	0.254
D(lnKOFGI(-1))	0.591∗∗∗	3.158		
D(lnKOFGI(-2))	0.783∗∗∗	4.274		

Note: ∗, ∗∗ and ∗∗∗ indicate 10 %, 5 % and 1 % significance levels, respectively.

**Table 6:** FMOLS regression results.

Variable	Temperature model		Precipitation model	
	Coefficient	t-Statistic	Coefficient	t-Statistic
lnAT	−0.519∗∗∗	−3.669		
lnPR			−0.015	−0.175
lnGCF	0.127∗∗	2.384	0.143∗∗∗	3.349
lnLABOUR	0.153∗∗	2.708	0.269∗∗∗	3.612
lnKOFGI	0.366∗∗	2.307	0.118	1.034
lnCPS	0.102∗∗∗	3.359	0.012∗∗	2.105
R-squared	0.966		0.738	
Adjusted R-squared	0.957		0.708	

Note: ∗, ∗∗ and ∗∗∗ indicate 10 %, 5 % and 1 % significance levels, respectively.

As displayed in Table 4, the log of AT is negatively signed and statistically significant at the 5 % level, which implies that temperature exerts a negative impact on the GDP per capita of Nigeria in the long run. Specifically, this result demonstrates that a percentage rise in average temperature is associated with a 0.67 decline in economic growth, ceteris paribus. This result suggests that climate change is an important influencer of economic growth in Nigeria. As argued in previous studies, temperature level could influence the country's economic growth through its impact on agricultural productivity. It has been reported that a warmer climate due to rising temperature has increased incidences of drought, with a consequent decline in the agricultural sector's contribution to the GDP [74,75]. Moreover [76], showed how heat stress, especially during the hot summer, causes huge revenue loss of diary output and revenue in the semi-arid regions of Nigeria, thereby reducing their contributions to the national output.

Labour productivity and its contribution to the GDP is also negatively impacted when temperature rises, as the prevalence of diarrhoea reportedly increases in the face of rising temperature [77]. As such, increases in temperature impede learning and have a negative effect on workforce health. These have the combined effect of lowering labour productivity and long-run economic growth. This research outcome is consistent with previous studies by Ref. [31] for sub-Saharan Africa and [71], which found that an increase in temperature is linked to a decline in economic growth in the Middle East and North Africa (MENA) countries.

In the second model, the log of PR is negative and weakly significant at the 10 % level. This indicates that precipitation negatively affects the GDP per capita in the long run. Specifically, a percentage increase in precipitation is associated with a 0.23 % decline in economic growth, ceteris paribus. This result suggests that precipitation is a key determinant of Nigeria's long-run economic growth level. Though an adequate level of precipitation is crucial for agricultural productivity, water resources and hydropower energy production, increased precipitation, particularly in the form of rainfall and floods, can damage infrastructure, disrupt transportation networks, and lead to significant economic losses [78,79]. This research outcome conforms to the finding of [71] that precipitation harms economic growth in MENA countries.

In the temperature model, the coefficient of capital stock is also positive and statistically significant at 1 % but negative and insignificant in the precipitation model. The results of the temperature model show that capital stock has a positive impact on economic growth in the long run. This result implies that capital stock is a key driver of economic growth in Nigeria. It also reinforces the premise of the endogenous growth theory that capital plays a key role in the attainment of long-run growth [80]. Labour force participation is also found crucial for economic growth based on the results from both models, as the coefficient of labour is positive and significant at 1 % and 5 % in the temperature and precipitation models, respectively. This result supports the previous finding by Ref. [81] that a long-run association exists between labour force participation and economic growth in Nigeria and that causality runs from the former to the latter.

The KOF Globalisation Index has been found to exert a positive impact on economic growth in the temperature model. It reveals that a percentage increase in globalisation intensity is associated with a 0.59 % increase in long-run economic growth, ceteris paribus. This result reinforces a previous finding by Ref. [82] that about 98 % of Nigeria's growth performance may be attributed to the dynamics of globalisation. However, this variable is statistically insignificant in the Precipitation model. Financial development is found to be statistically significant and positive according to the Temperature model. This indicates that financial development is an important driver of economic growth in Nigeria in the long run. This suggests that as the country's financial sector develops, resources can be more effectively allocated to useful ends, leading to the financing of creative and entrepreneurial ideas. This result supports the conclusion made by previous studies [83,84] that financial development positively and strongly influences economic growth.

The long-run estimates of the FMOLS regression are provided in Table 6. The results are predominantly the same as in the ARDL estimates, except for the coefficient of precipitation, which is insignificant. This is unsurprising as the ARDL counterpart is weakly significant at the 10 % level. This could only point to the fact that the precipitation analysis should be taken with a pinch of salt. As for the remaining estimates, the same interpretations and discussions as in the ARDL estimates are applicable.

The short-run results are provided in the lower compartment of Table 5. In each of the two models, the error correction term (ECT) yields a negative and significant coefficient at 1 % level of significance. This confirms the existence of a long-run relationship between the variables, which has already been established in this study via ARDL bound testing. This parameter controls how quickly a stable and dynamic equilibrium returns over time after distortions and divergences brought on by shocks. The coefficients of ECT, as reported in the table, suggest that following a shock along the long-run path via the short-run distortions, convergence to equilibrium is attained within a year at the speed of 11.1 % and 52.0 % in the Temperature model and Precipitation model, respectively.

Regarding the short-run estimates, the coefficients of lag 0 and lag 1 of the log of AT are both positive and significant at 5 % and 1 %, respectively. Adding the two coefficients (lag 0 and lag 1) together results in a positive coefficient (0.723). Furthermore, the Wald test at a 5 % level of significance indicates that it is jointly significant. As such, this result confirms that temperature positively impacts Nigeria's economic growth in the short run. This is opposed to the long-run result that indicates a negative impact of temperature on economic growth. One plausible explanation for the differently signed long-run and short-run coefficients could be that a minimum temperature level is required for crop and livestock yields in the agricultural sector. This minimum level has not yet been attained in the short run but has already exceeded it in the long run. This argument supports the intrinsic nonlinearity of temperature and economic growth, as [31] demonstrated.

In the case of precipitation, the coefficient of lag 0 of the log of PR is positive and insignificant, while those of lag 1 and lag 2 are positive and significant at the 1 % level. The sum of lags 0, 1 and 2 yields a positive coefficient (0.632), and its Wald test suggests a joint significance at 5 %. This result indicates that in the short run, precipitation enhances economic growth. It further shows that adequate rainfall is key to avoiding droughts, crop failures and declining agricultural output in a country like Nigeria, where farming is relied upon for livelihood by part of the population. Sufficient precipitation is also crucial for maintaining water resources, including reservoirs and groundwater, which are vital for various sectors such as energy production (hydropower), industry, and household consumption. All these contribute to economic growth.

In the temperature model, the coefficients of lag 0 and lag 1 of GCF are positive and significant, implying that capital stock influences economic growth in the short run. This corroborates the proposition put out by the endogenous growth model that capital is a crucial component of growth. However, this variable and all other variables are not significant in the precipitation model. The lag 0 of the log of the KOF globalisation index is negative and significant at the 1 % level, while lags 1 and 2 of the Index are positive and significant at 1 %. The coefficient sum of the three lags (lags 0, 1 and 2) is positive (0.788), and the Wald test indicates significance at 5 %. This indicates that globalisation enhances economic growth in the long run.

### 4.4 Distributional impact of climate change on Nigeria's economic growth

In this section, we examined the impact of climate change (average temperature and precipitation) across the conditional distribution of economic growth in Nigeria. Limiting the interpretation to the variables of interest, the results shown in Table 7 indicate that average temperature and precipitation negatively and statistically significantly impact economic growth in Nigeria. However, the magnitude of these climate change variables' impact differs across economic growth quantiles. For instance, the elasticity of average temperature on economic growth increases from −0.311 in the 25th quartile to −0.939 in the 50th quartile and −1.091 in the 75th quartile. However, the magnitude of precipitation reduced from −0.290 in the 25th quartile to −0.203 in the 50th quartile and −0.279 in the 75th quartile. However, the short-run impact of climate change on economic growth is positive and unanimous across the condition distribution of economic growth in Nigeria. Specifically, the one lag of average temperature impact ranged from 0.274 in the 25th quartile to 0.635 in the 50th quartile and 0.868 in the 75th quartile. Additionally, precipitation's impact also ranged from 0.591 in the 25th quartile to 0.518 in the 50th quartile and 0.388 in the 75th quartile.

**Table 7:** Quantile ARDL model.

Variable	Temperature model			Precipitation model		
	Q = 0.25	Q = 0.50	Q = 0.75	Q = 0.25	Q = 0.50	Q = 0.75
**Long-run results:**						
lnAT	−0.311	−0.939∗∗	−1.091∗∗∗			
	(0.704)	(0.465)	(0.450)			
lnPR				−0.290∗∗	−0.203	−0.279
				(0.149)	(0.221)	(0.233)
Control variables	Yes	Yes	Yes	Yes	Yes	Yes
**Short-run results:**						
ECT	0.017	−0.009∗∗	−0.186∗∗	−0.065	−0.086	−0.066
	(0.134)	(0.203)	(0.273)	(0.111)	(0.201)	(0.161)
D(lnAT)	0.277	0.314	0.227			
	(0.194)	(0.285)	(0.208)			
D(lnAT(-1))	0.274	0.635∗∗	0.868∗			
	(0.385)	(0.275)	(0.481)			
D(lnPR)				0.137	0.108	0.007
				(0.092)	(0.129)	(0.184)
D(lnPR(-1))				0.591∗∗	0.518∗∗∗	0.388
				(0.392)	(0.212)	(0.340)
D(lnPR(-2))				0.121	0.390∗∗∗	0.224
				(0.144)	(0.167)	(0.189)
Control variables	Yes	Yes	Yes	Yes	Yes	Yes
Pseudo R-squared	0.578	0.504	0.489	0.545	0.332	0.218

Note: ∗, ∗∗ and ∗∗∗ indicate 10 %, 5 % and 1 % significance levels, respectively. Huber Sandwich Standard Errors are in brackets.

One resounding message from this result is that the QARDL results authenticate the empirical outcomes of the ARDL and FMOLS models, which show that climate change negatively impacts economic growth in the long run while the short-run impact is positive. We believe that in the short run, the impact of climate change may increase economic activity in Nigeria, as there is a surge in economic activity related to reconstruction and recovery efforts after extreme weather events. Another example could be that sudden changes in weather patterns or resource availability may create demand for specific industries. For example, warmer temperatures might increase demand for air conditioning or certain crops, providing short-term economic opportunities.

A plausible explanation for the long-run negative impact is that changes in temperature and precipitation patterns can adversely affect agricultural productivity, leading to reduced crop yields. This can impact food security, increase prices, and have long-term consequences on the agricultural sector.^5^ Similarly, climate change can also contribute to the incidence of heat-related disease, and the long-term health impact can reduce the productivity of labour.

### 4.5 Diagnostics

In this section, the validity of the estimates is verified through a few diagnostic tests, and the results are presented in Table 8. For both the temperature and precipitation models, the error terms are indicated to be normally distributed, according to the Jaque-Bera statistics, as the probability is higher than 0.05 in each case. Additionally, the Breusch-Pagan-Godfrey test for heteroskedasticity in the two models supports the lack of heteroskedasticity, while serial correlation tests suggest the absence of serial correlation in the two models. Furthermore, Ramsey's RESET statistics support the model specification of temperature and precipitation models, indicating that the models' functional forms are correctly specified. Overall, the results of this variety of tests show that the estimated parameters are reliable.

**Table 8:** ARDL regression diagnostic tests.

Test	Temperature model	Precipitation model
	Probability	Probability
Jaque-Bera normality	0.839	0.817
Serial correlation LM	0.771	0.715
Heteroskedasticity	0.823	0.854
Ramsey Specification	0.1777	0.4200

**Fig. 4:**
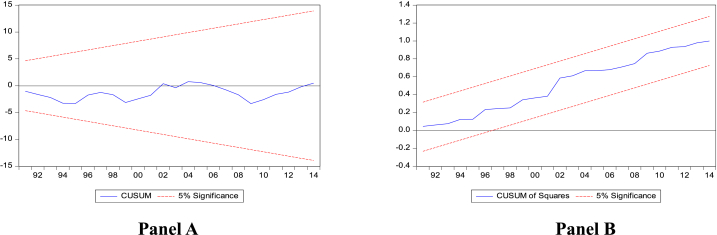
CUSUM and CUSUM of squares plots of recursive residuals (temperature model).

**Fig. 5:**
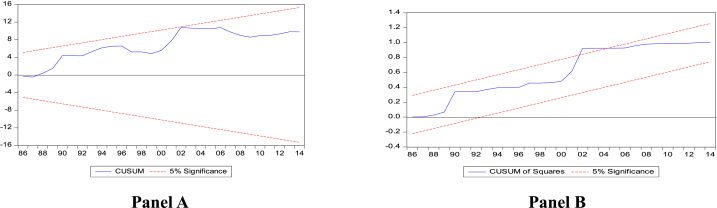
CUSUM and CUSUM of squares plots of recursive residuals (precipitation model).

The cumulative sum (CUSUM) and cumulative sum of squares (CUSUMSQ) to the recursive residuals estimated are used to confirm the stability of the error correction version of ARDL parameters in the two models. The results of the temperature model are presented in Fig. 4 (Panels A and B), while those of the precipitation model are presented in Fig. 5 (Panels A and B). As a result of the lines being within 5 % of the crucial boundaries (except for the precipitation model's CUSUMSQ, where the line very slightly falls outside the line),^6^ CUSUM and CUSUMSQ validate the stability of the parameters and the model estimated, as suggested by Ref. [85]. The correlation matrix is presented in Table A1 in the Appendix. The results presented in the table indicate that none of the main explanatory variables (temperature precipitation, labour, globalisation, gross capital formation, and financial development) has a correlation coefficient over 0.7, excluding the possibility of significant multicollinearity.

### 4.6 Time-varying causality between climate change and economic growth in Nigeria

**Fig. 6:**
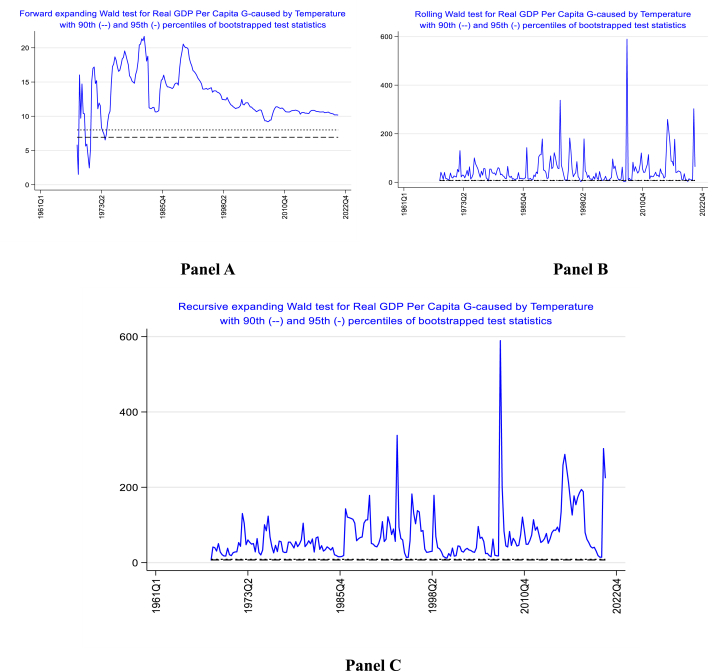
Time-varying causality between average temperature and real GDP per capita in Nigeria.

In this section, we investigate the existence of causality between climate change and economic growth in Nigeria using the LA-VAR model that accounts for heteroskedasticity [38]. The minimum window size is set to 30 (7.5 quarters). The lag length of 6 is selected using the Akaike information and Schwarz information criteria. The results of the forward algorithm in Fig. 6A indicate that the causal effects have changed considerably over the years. In some of these years, causal effects are detected where the Wald tests are above the critical level of significance (90th and 95th percentile of bootstrapped test statistics). However, in some years, causations are not detected (Wald tests are below the critical level). Furthermore, our empirical results indicate very strong causation from climate change to economic growth in 1980–1982 and 1990 to 1991. This episode coincides with when Nigeria experienced a high record of 25.75 °C in 1980 and 25.69 °C in 1990.

Furthermore, the rolling and recursive evolving procedures presented in Panel B and C of Fig. 6 identify causality from average temperature to GDP growth in Nigeria throughout the entire period since the Wald tests are above the critical significance level at the 90th and 95th percentile of bootstrapped test statistics. However, one key feature of the figures is that 1993, 2007, 2016, and 2021 appear to be the years with the strongest causation from climate change to economic growth. Coincidentally, one of these periods coincides with one of the hottest periods in Nigeria's history (i.e., 2021, with an average temperature of 25.82 °C).

**Fig. A1:**
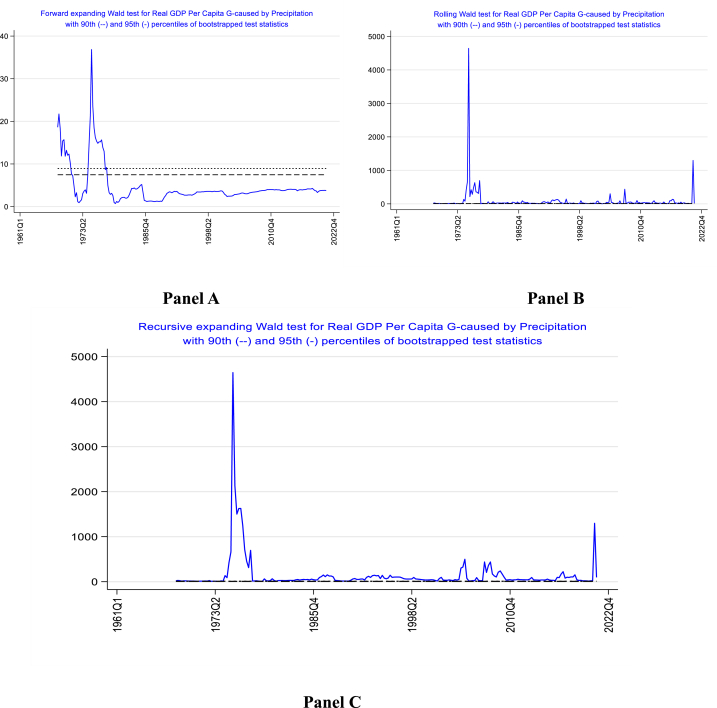
Time-varying causality between average precipitation and real GDP per capita in.

The time-varying causality between climate change and economic growth is extended to an alternative climate change measure, precipitation, using the forward, rolling, and recursive evolving procedures. The minimum window size is set to 30 (7.5 quarters), and the lag length of 6 is selected using the Akaike information and Schwarz information criteria. The empirical outcome presented in Panel A, B and C of Figure A1 indicates variability in causation throughout the years. In some of these years, causal effects are detected where the Wald tests are above the critical level of significance (90th and 95th percentile of bootstrapped test statistics). However, causation is not detected in some years.

Generally, we can conclude that climate change causes economic growth in Nigeria. This connotes that increased temperatures, changes in precipitation patterns, and more frequent and severe extreme weather events have a ripple effect on the Nigerian economy.

## 5 Conclusion and policy prescriptions

The hazards associated with Nigeria's fragility are being exacerbated by the effects of climate change on its socioeconomic and environmental systems. The challenges confronting local communities are worsened by extreme weather patterns, which include shorter, more intense rainy seasons and hotter, longer dry seasons. Large tracts of land in northern Nigeria are now unusable due to excessive grazing and extensive farming. Higher-intensity, unpredictable rainfall in southern Nigeria is displacing populations and causing agricultural loss. Given the nation's rapidly expanding population, the country's depleting environmental resources provide a significant threat to food security. Nigeria is ranked as the sixth least prepared country and the 53rd most vulnerable country in the world.^7^

This study examined the relationship between climate change and economic growth in Nigeria for the period 1980 to 2020. To enrich the depth of this analysis, this study considers average temperature and precipitation as central indicators of climate change. We employed cutting-edge econometric approaches comprising Autoregressive Distributed Lag, fully modified least squares, innovative Quantile Autoregressive Distributed Lag, and time-varying causality. The short-run estimates suggest that climate change positively influences economic growth. However, the long-run estimates derived from both the ARDL and FMOLS regression models revealed more nuanced insights about the nexus between climate change and economic growth. Specifically, the results of average temperature (AT) and precipitation (PR) reveal a negative and statistically significant impact on GDP per capita, thereby confirming the susceptibility of Nigeria's economic landscape to climate change.

Similarly, the QARDL results reveal that in the majority of instances, economic growth experiences a decline with increasing temperatures, except for the lowest quantiles, whilst precipitation exerts a negative influence among the higher quantiles. The time-varying causality also indicates a unidirectional causality between climate change and economic growth.

The empirical outcome of this study has produced several policy imperatives for the Nigerian governments, policymakers, and crucial stakeholders: (1) it is important that the Nigerian government provide financial incentives for businesses and investors engaged in environmentally friendly projects. Doing this would enhance ecologically sustainable GDP benefits. Similarly, the utilisation and issuance of green bonds for ecologically sustainable development should also be promoted. (2) climate change pedagogy should be included in the formal education system to build a future generation with a strong environmental consciousness and launch campaigns to raise awareness about climate change and its impacts, and (3) it is also imperative for the government to investment in renewable energy; Nigerian government should provide financial incentives and subsidies to encourage the adoption of renewable energy sources such as solar, wind, and hydropower. This study has some shortcomings which can be addressed in future research. Other intermittent or mediating variables, such as macroeconomic policies, have been adjudged to be crucial in mitigating the impact of climate change. Another limitation of this study is the potential influence of structural breaks in the CUSUM square statistic. Structural breaks, which refer to sudden changes in the data-generating process, can cause the variance of the time series to fluctuate significantly. This fluctuation can lead to the CUSUM square statistic falling outside its control limits, potentially affecting the reliability of our findings. Future studies could employ methods that accommodate structural breaks. Future research could also look at the role of fiscal or monetary policy on the impact of climate change and economic outcomes in Nigeria.

## CRediT authorship contribution statement

**Sodiq Arogundade:** Validation, Methodology, Investigation, Formal analysis, Data curation, Conceptualization. **Adewale Samuel Hassan:** Methodology, Investigation, Data curation, Conceptualization. **Biyase Mduduzi:** Investigation.

## Data availability statement

The data supporting this study's findings are available on request from the corresponding author.

## Funding

Not applicable.

## Declaration of competing interest

The authors declare that they have no known competing financial interests or personal relationships that could have appeared to influence the work reported in this paper.

## Figures and Tables

**Table A1:** Matrix of correlation.

	TAS	PR	L	GCF2	KOFGI	CPS
TAS	1.000	0.297	0.374	0.086	0.352	0.234
PR	0.297	1.000	0.163	0.153	0.188	0.205
L	0.374	0.163	1.000	0.579	0.664	0.594
GCF2	0.086	0.153	0.579	1.000	0.485	0.367
KOFGI	0.352	0.188	0.664	0.485	1.000	0.676
CPS	0.234	0.205	0.594	0.367	0.676	1.000

## Footnotes

1 https://www.ucl.ac.uk/news/2009/may/climate-change-biggest-global-health-threat-21st-century.

2 https://press.un.org/en/2021/sc14445.doc.htm.

3 26^th^ meeting of the Conference of Parties.

4 https://climateknowledgeportal.worldbank.org/country/nigeria/climate-data-historical.

5 This is also crucial since the agricultural sector contributed an estimated value of 25.88 % to real GDP in 2022 (See Ref. [86]).

6 We believe this might be because of a change in precipitation in the specific period.

7 See Ref. [87] for more.

## References

[1] Khan Z., Ali S., Dong K., Li R.Y.M. (2021). How does fiscal decentralization affect CO2 emissions? The roles of institutions and human capital. Energy Econ..

[2] Skytt T., Nielsen S.N., Jonsson B.G. (2020). Global warming potential and absolute global temperature change potential from carbon dioxide and methane fluxes as indicators of regional sustainability – a case study of Jämtland, Sweden. Ecol. Indicat..

[3] Buhaug H., Benjaminsen T.A., Gilmore E.A., Hendrix C.S. (2023). Climate-driven risks to peace over the 21st century. Clim Risk Manag.

[4] Chivangulula F.M., Amraoui M., Pereira M.G. (2023). The drought regime in southern Africa: a systematic review. Climate.

[5] Lamperti F., Bosetti V., Roventini A., Tavoni M., Treibich T. (2021). Three green financial policies to address climate risks. J. Financ. Stabil..

[6] Oberoi S.S., Banerjee S. (2024). Effect of climate change, damage to environment and human development index on economic growth in Nigeria. African Journal of Business and Economic Research.

[7] Sasai F., Roncal-Jimenez C., Rogers K., Sato Y., Brown J.M., Glaser J., Garcia G., Sanchez-Lozada L.G., Rodriguez-Iturbe B., Dawson J.B., Sorensen C., Hernando A.A., Gonzalez-Quiroz M., Lanaspa M., Newman L.S., Johnson R.J. (2023). Climate change and nephrology. Nephrol. Dial. Transplant..

[8] Zhao Y., Liu S. (2023). Effects of climate change on economic growth: a perspective of the heterogeneous climate regions in Africa. Sustainability.

[9] Ojo T.O., Baiyegunhi L.J.S. (2021). Climate change perception and its impact on net farm income of smallholder rice farmers in South-West, Nigeria. J. Clean. Prod..

[10] (2023). Climate Change 2021 – the Physical Science Basis.

[11] Glasgow Climate Change Conference – October-November 2021 | UNFCCC, (n.d.) https://unfccc.int/conference/glasgow-climate-change-conference-october-november-2021 (accessed September 26, 2024).

[12] Stern N. (2021). A time for action on climate change and a time for change in economics. http://www.cccep.ac.uk.

[13] Fao, FAO’S WORK ON CLIMATE CHANGE n.d.

[14] WHO (2021).

[15] S. Hallegatte, M. Bangalore, L. Bonzanigo, M. Fay, T. Kane, U. Narloch, J. Rozenberg, D. Treguer, A. Vogt-Schilb, SHOCK WAVES Climate Change and Development Series Managing the Impacts of Climate Change on Poverty,n.d.

[16] Nigeria Overview: Development news, research, data | World Bank, (n.d.). https://www.worldbank.org/en/country/nigeria/overview (accessed September 26, 2024).

[17] A fresh perspective on Nigeria's economic growth | McKinsey,(n.d.). https://www.mckinsey.com/featured-insights/middle-east-and-africa/microregional-data-uncover-a-richer-picture-of-development-in-nigeria. (accessed September 26, 2024).

[18] Renaud F.G., Birkmann J., Damm M., Gallopín G.C. (2010). Understanding multiple thresholds of coupled social–ecological systems exposed to natural hazards as external shocks. Nat. Hazards.

[19] Getirana A., Libonati R., Cataldi M. (2021). Brazil is in water crisis — it needs a drought plan. Nature.

[20] 5 natural disasters that beg for climate action | Oxfam International,(n.d.). https://www.oxfam.org/en/5-natural-disasters-beg-climate-action (accessed September 26, 2024).

[21] Zhu T., Fonseca De Lima C.F., De Smet I. (2021). The heat is on: how crop growth, development, and yield respond to high temperature. J. Exp. Bot..

[22] bp, Statistical Review of World Energy 2022,n.d.

[23] Ayanlade A., Proske U. (2016). Assessing wetland degradation and loss of ecosystem services in the Niger Delta, Nigeria. Mar. Freshw. Res..

[24] Adekola O., Mitchell G., Grainger A. (2015). Inequality and ecosystem services: the value and social distribution of Niger Delta wetland services. Ecosyst. Serv..

[25] R. Dauda, Climate change and food security in Nigeria faculty member, NESG Non-Residential Fellowship Programme E P R J O U R N A L H 2 ’ 2 0 2 3 n.d.

[26] Nigeria Climate Change Country Profile | U.S. Agency for International Development,(n.d.). https://www.usaid.gov/climate/country-profiles/nigeria (accessed September 26, 2024).

[27] Nigeria prioritizes climate action to mitigate natural disasters | Africa Renewal,(n.d.). https://www.un.org/africarenewal/magazine/august-2023/nigeria-prioritizes-climate-action-mitigate-natural-disasters (accessed September 26, 2024).

[28] Kiley M.T. (2024). Growth at risk from climate change. Econ. Inq..

[29] Abidoye B.O., Odusola A.F. (2015). Climate change and economic growth in Africa: an econometric analysis. J. Afr. Econ..

[30] Farajzadeh Z., Ghorbanian E., Tarazkar M.H. (2022). The shocks of climate change on economic growth in developing economies: evidence from Iran. J. Clean. Prod..

[31] Alagidede P., Adu G., Frimpong P.B. (2014). The effect of climate change on economic growth: evidence from Sub-Saharan Africa.

[32] Baarsch F., Granadillos J.R., Hare W., Knaus M., Krapp M., Schaeffer M., Lotze-Campen H. (2020). The impact of climate change on incomes and convergence in Africa. World Dev..

[33] Burke M., Davis W.M., Diffenbaugh N.S. (2018). Large potential reduction in economic damages under UN mitigation targets. Nature.

[34] Tol R.S.J. (2024). A meta-analysis of the total economic impact of climate change. Energy Pol..

[35] Cho J.S., Kim T., Shin Y. (2015). Quantile cointegration in the autoregressive distributed-lag modeling framework. J. Econom..

[36] Toda H.Y., Yamamoto T. (1995). Statistical inference in vector autoregressions with possibly integrated processes. J. Econom..

[37] Dolado J.J., Lütkepohl H. (1996). Making wald tests work for cointegrated VAR systems. Econom. Rev..

[38] Shi S., Hurn S., Phillips P.C.B. (2020). Causal change detection in possibly integrated systems: revisiting the money–income relationship. J. Financ. Econom..

[39] Chapter Climate Change 2014 Synthesis Report Summary for Policymakers Summary for Policymakers,n.d.

[40] World Development Indicators | DataBank (n.d.). https://databank.worldbank.org/source/world-development-indicators (accessed September 26, 2024).

[41] Nigeria - Climatology | Climate Change Knowledge Portal,(n.d.). https://climateknowledgeportal.worldbank.org/country/nigeria/climate-data-historical (accessed September 26, 2024).

[42] How Climate Change Is Disrupting the Global Supply Chain - Yale E360,(n.d.). https://e360.yale.edu/features/how-climate-change-is-disrupting-the-global-supply-chain (accessed September 27, 2024).

[43] Crimmins A., Balbus J., Gamble J.L., Beard C.B., Bell J.E., Dodgen D., Eisen R.J., Fann N., Hawkins M.D., Herring S.C., Jantarasami L., Mills D.M., Saha S., Sarofim M.C., Trtanj J., Ziska L. (2016). The impacts of climate change on human health in the United States: a scientific assessment, Washington, DC.

[44] Caruso R., Petrarca I., Ricciuti R. (2016). Climate change, rice crops, and violence. J. Peace Res..

[45] Marchiori L., Maystadt J.-F., Schumacher I. (2012). The impact of weather anomalies on migration in sub-Saharan Africa. J. Environ. Econ. Manag..

[46] UNSDG | Climate crisis in Nigeria: The UN fosters dialogue between farmers and cattle herders over shrinking land, (n.d.) https://unsdg.un.org/latest/stories/climate-crisis-nigeria-un-fosters-dialogue-between-farmers-and-cattle-herders-over (accessed September 27, 2024).

[47] Okunade S.K., Kohon H.S. (2023). Contemporary Issues on Governance, Conflict and Security in Africa.

[48] Dasgupta S., van Maanen N., Gosling S.N., Piontek F., Otto C., Schleussner C.F. (2021). Effects of climate change on combined labour productivity and supply: an empirical, multi-model study. Lancet Planet. Health.

[49] PESETA III: Climate change impacts on labour productivity, (n.d.).https://doi.org/10.2760/07911.

[50] Arogundade S., Mduduzi B., Hassan A.S. (2022). Spatial impact of foreign direct investment on ecological footprint in Africa. Environ. Sci. Pollut. Control Ser..

[51] Pata U.K., Samour A. (2022). Do renewable and nuclear energy enhance environmental quality in France? A new EKC approach with the load capacity factor. Prog. Nucl. Energy.

[52] Kalim R., Ul-Durar S., Iqbal M., Arshed N., Shahbaz M. (2024). Role of knowledge economy in managing demand-based environmental Kuznets Curve. Geosci. Front..

[53] Pata U.K., Samour A. (2022). Do renewable and nuclear energy enhance environmental quality in France? A new EKC approach with the load capacity factor. Prog. Nucl. Energy.

[54] Hassan A.S., Mhlanga D. (2023). The asymmetric effect of oil price on ecological footprint: evidence from oil-producing African countries. Sustainable Energy Research.

[55] Hassan A.S. (2022). Coal mining and environmental sustainability in South Africa: do institutions matter?. Environ. Sci. Pollut. Control Ser..

[56] Hassan A.S. (2023). Modeling the linkage between coal mining and ecological footprint in South Africa: does technological innovation matter?. Mineral Economics.

[57] Arogundade S., Hassan A.S., Bila S. (2022). Diaspora income, financial development and ecological footprint in Africa. Int. J. Sustain. Dev. World Ecol..

[58] Yang Y., Guo H., Chen L., Liu X., Gu M., Ke X. (2019). Regional analysis of the green development level differences in Chinese mineral resource-based cities. Resour. Pol..

[59] Mohammed Kamel Si, Korkut Pata U. (2024). Linking the utilization of mineral resources and climate change: a novel approach with frequency domain analysis. Geosci. Front..

[60] Schlenker W., Auffhammer M. (2018). The cost of a warming climate. Nature.

[61] Adom P.K., Amoani S. (2021). The role of climate adaptation readiness in economic growth and climate change relationship: an analysis of the output/income and productivity/institution channels. J. Environ. Manag..

[62] Solow R.M. (1956). A contribution to the theory of economic growth. Q. J. Econ..

[63] Ali S.N. (2012). Climate change and economic growth in a rain-fed economy: how much does rainfall variability cost Ethiopia?. SSRN Electron. J..

[64] Dell M., Jones B.F., Olken B.A. (2009). Temperature and income: reconciling new cross-sectional and panel estimates. Am. Econ. Rev..

[65] Berihun D., Van Steven P. (2022). Climate variability and macroeconomic output in Ethiopia: the analysis of nexus and impact via asymmetric autoregressive distributive lag cointegration method. Environ. Dev. Sustain..

[66] Pesaran M.H., Shin Y., Smith R.J. (2001). Bounds testing approaches to the analysis of level relationships. J. Appl. Econom..

[67] Narayan P.K., Narayan S. (2010). Carbon dioxide emissions and economic growth: panel data evidence from developing countries. Energy Pol..

[68] Ozcan B. (2013). The nexus between carbon emissions, energy consumption and economic growth in Middle East countries: a panel data analysis. Energy Pol..

[69] Phillips P.C.B., Hansen B.E. (1990). Statistical inference in instrumental variables regression with I(1) processes. Rev. Econ. Stud..

[70] Hamit-Haggar M. (2012). Greenhouse gas emissions, energy consumption and economic growth: a panel cointegration analysis from Canadian industrial sector perspective. Energy Econ..

[71] Meyghani S., Khodaparast Mashhadi M., Salehnia N. (2023). Long-term effects of temperature and precipitation on economic growth of selected MENA region countries. Environ. Dev. Sustain..

[72] 2009: Second warmest year on record; end of warmest decade – Climate Change: Vital Signs of the Planet,(n.d.). https://climate.nasa.gov/news/249/2009-second-warmest-year-on-record-end-of-warmest-decade/(accessed September 28, 2024).

[73] Zivot E., Andrews D.W.K. (1992). Further evidence on the great crash, the oil-price shock, and the unit-root hypothesis. J. Bus. Econ. Stat..

[74] Nhemachena C., Nhamo L., Matchaya G., Nhemachena C.R., Muchara B., Karuaihe S.T., Mpandeli S. (2020). Climate change impacts on water and agriculture sectors in southern Africa: threats and opportunities for sustainable development. Water.

[75] Maponya P., Mpandeli S. (2012). Climate change and agricultural production in South Africa: impacts and adaptation options. J. Agric. Sci..

[76] Ogundeji A.A., Lakew H., Tesfuhuney W., Lombard W. (2021). Influence of heat stress on milk production and its financial implications in semi-arid areas of South Africa. Heliyon.

[77] Bandyopadhyay S., Kanji S., Wang L. (2012). The impact of rainfall and temperature variation on diarrheal prevalence in Sub-Saharan Africa. Appl. Geogr..

[78] Dube K., Nhamo G., Chikodzi D. (2022). Flooding trends and their impacts on coastal communities of Western Cape Province, South Africa. Geojournal.

[79] Musyoki A., Thifhulufhelwi R., Murungweni F.M. (2016). The impact of and responses to flooding in Thulamela Municipality, Limpopo Province, South Africa, Jàmbá. Journal of Disaster Risk Studies.

[80] Lucas R.E. (1988). On the mechanics of economic development. J. Monetary Econ..

[81] Yakubu M.M., Akanegbu B.N. (2020).

[82] Globalisation and Economic Growth in South Africa: Do We Benefit from Trade and Financial Liberalisation? 1 Elsabe Loots,.(n.d.).

[83] Law S.H., Kutan A.M., Naseem N.A.M. (2018). The role of institutions in finance curse: evidence from international data. J. Comp. Econ..

[84] Pan F., Yang B. (2019). Financial development and the geographies of startup cities: evidence from China. Small Bus. Econ..

[85] Bahmani-Oskooee M., Chi R., Ng W. (2002).

[86] Reports | National Bureau of Statistics,(n.d.). https://nigerianstat.gov.ng/elibrary/read/1241288 (accessed September 26, 2024).

[87] Country Index//Notre Dame Global Adaptation Initiative//University of Notre Dame(n.d.). https://gain.nd.edu/our-work/country-index/(accessed September 26, 2024).

